# Smoking load reduction is insufficient to downregulate miR-301b, a lung cancer promoter

**DOI:** 10.1038/s41598-020-78242-0

**Published:** 2020-12-03

**Authors:** Camila dos Santos Arcas, Hui Tzu Lin-Wang, Iracema Ioco Kikuchi Umeda, Márcio Gonçalves de Sousa, Daniela Mitiyo Odagiri Utiyama, Antonio de Padua Mansur, Mariangela Macchione, Mario Hiroyuki Hirata, Naomi Kondo Nakagawa

**Affiliations:** 1 Department of Physiotherapy, LIM-54, Faculdade de Medicina da Universidade de São Paulo, Av. Dr. Arnaldo, 455 room 1150, São Paulo, São Paulo 01246-930 Brazil; 2 Dante Pazzanese Institute of Cardiology São Paulo State, São Paulo, Brazil; 3 Medicine Rehabilitation Lucy Montoro Institute, Faculdade de Medicina da Universidade de São Paulo, São Paulo, Brazil; 4 Department of Cardiopneumology, Faculdade de Medicina da Universidade de São Paulo, São Paulo, Brazil; 5 Department of Pathology, LIM05, Faculdade de Medicina da Universidade de São Paulo, São Paulo, Brazil; 6 Department of Clinical and Toxicological Analysis, School of Pharmaceutical Sciences, Universidade de São Paulo, São Paulo, Brazil

**Keywords:** Lung cancer, miRNAs, Biomarkers

## Abstract

Several circulating miRNAs identified in the plasma of smokers have been implicated as promoters of nasopharyngeal and lung carcinoma. To investigate the plasma profile of miRNAs in subjects who reduces the number of smoked cigarettes and who quit after six months. We accompanied 28 individuals enrolled in a Smoking Cessation Program over 6 months. At Baseline, clinical characteristics, co-morbidities, and smoking history were similar among subjects. After 6 months, two groups were defined: who successfully quitted smoking (named “quitters”, n = 18, mean age 57 years, 11 male) and who reduced the number of cigarettes smoked (20–90%) but failed to quit smoking (named “smokers”, n = 10, mean age 52 years, 3 male). No significant clinical changes were observed between groups at baseline and after a 6-month period, however, quitters showed significant downregulations in seven miRNAs at baseline: miR-17 (− 2.90-fold, p = 0.029), miR-20a (− 3.80-fold, p = 0.021); miR-20b (− 4.71-fold, p = 0.027); miR-30a (− 3.95-fold, p = 0.024); miR-93 (− 3.63-fold, p = 0.022); miR-125a (− 1.70-fold, p = 0.038); and miR-195 (− 5.37-fold, p = 0.002), and after a 6-month period in 6 miRNAs: miR-17 (− 5.30-fold, p = 0.012), miR-20a (− 2.04-fold, p = 0.017), miR-20b (− 5.44-fold, p = 0.017), miR-93 (− 4.00-fold, p = 0.041), miR-101 (− 4.82-fold, p = 0.047) and miR-125b (− 3.65-fold, p = 0.025). Using time comparisons, only quitters had significant downregulation in miR-301b (− 2.29-fold, p = 0.038) after 6-month. Reductions in the number of smoked cigarettes was insufficient to change the plasma profile of miRNA after 6 months. Only quitting smoking (100% reduction) significantly downregulated miR-301b related to hypoxic conditions, promotion of cell proliferation, decreases in apoptosis, cancer development, and progression as increases in radiotherapy and chemotherapy resistance.

## Introduction

Tobacco smoking is a significant risk factor for lung diseases, coronary heart disease, stroke, cancer and death^1^. Tobacco smoking can promote cell hyperplasia mutations and also tumorigenesis^2,3^. Tobacco smoking is decreasing worldwide, however, prevalence is still high in men (21%) and also in women (6%)^4^.

Smoking cessation is the primary strategy to refrain from respiratory disease development and/or progression^5–7^. However, rates of smoking cessation adherence vary over time and are associated with several biological, psychological and social aspects^8,9^. A high- quality study showed that approximately 53% are quitters at one-month, and only 14.6% are still abstinents after 12 months^10^. In this scenario, reducing the number of cigarettes per day could be raised as a bridge to smoking cessation or a tobacco smoking harm control for inveterate smokers^11,12^.

MiRNAs play a crucial role in several biological and pathological processes as various responses to therapeutics. They act as regulators of gene expression by inhibiting protein translation or mRNA degradation. Smoking can induce miRNAs expression dysregulation in the airways and lungs^3,13^. Some miRNAs have been described as biomarkers for early diagnosis of COPD and lung cancer in smokers^14,15^.

Smoking cessation leads to the downregulation of some miRNAs related to inflammation, immune response, oncogenesis, and apoptosis. The aim of the present study was to focus on the plasma miRNA profile of subjects enrolled in a smoking cessation program to investigate whether profiles were similar between who successfully quitted smoking and who failed but significantly reduced the number of cigarettes smoked per day after 6 months.

## Material and methods

This longitudinal study was approved by the Ethical Committee of the University of São Paulo Medical School, São Paulo, Brazil (CAAE: 33427014.0.0000.0065) and was conducted according to the Helsinki declaration. The inclusion criteria were both sexes, aged between 18 and 70 years, that were enrolled in the Smoking Cessation Program at Institute Dante Pazzanese of Cardiology State of São Paulo, and subjects that agreed with the written informed consent to participate in the present study. The exclusion criteria were respiratory infection in the 30 days and previous nasopharynx surgery.

Our smoking cessation program followed the Guidelines of the National Institute of Cancer (INCA) for Smoking Cessation Programs. All treatments were provided with no costs and included: (a) a psychological support offered in groups of subjects (1x/week) over at least 2 months, (b) pharmacological treatment that included bupropion 300 mg/day (twice/day) over 8 consecutive days followed by a reduction in buproprion to 150 mg/day (once/day) associated with nicotine replacement therapy over at least for 2 months. Demographic characteristics, clinical assessments, the exhaled carbon monoxide quantification (CO, ppm), the nasal mucociliary clearance and peripheral blood samples collection for miRNAs expression analysis were performed at Baseline and after 6 months.

Accordingly to our study protocol, data analysis would be performed after 6 months when subjects were divided into two groups: who adhered and successfully quitted smoking (named “quitters”) and who significantly reduced tobacco load but failed to quit smoking (called “smokers”). As pointed out by the Guidelines of the World Health Organization, a current smoker is a subject who had smoked ≥ 100 cigarettes and who currently smoke at least one cigarette per day^4^. To determine smoking status, exhaled CO was measured using a Micro CO Analyzer (Cardinal Health U.K. 232 Ltd., Chatham, UK), as reported by our group and others^5,10,16^. Briefly, subjects were asked to breathe through the analyzer, exhaling slowly from their total lung capacity with a constant expiratory flow of 5-6L/min over 10–15 s. Smoking cessation was defined as an exhaled CO < 6 ppm^17,18^.

To determine airway defense mechanism, we used the saccharin transit time test (STT) to assess nasal mucociliary clearance^19,20^. Briefly, we asked the individuals to avoid drinking coffee, black tea or alcohol on the test day. The subject was asked to sit in a chair and oriented to swallow freely, however, to avoid head movement, sneezing, or to sniff. After saccharin (25 µg) deposition in the free flow nostril, the individual reported the sweet taste as soon as it was perceived. The time (minutes) was recorded with the aid of a clock.

To determine lung function, we used spirometry (Koko model, PDS Instrumentation Inc., Colorado, USA), and tests were performed according to the recommendations of the American Thoracic Society and European Respiratory Society Task Force^21^. We recorded the absolute and percentage of predicted forced vital capacity (FVC), the forced expiratory volume in the first second (FEV_1_), the forced expiratory flow between 25 and 75% of the FVC (FEF_25-75%_) and the absolute value of the FEV_1_/FVC ratio. Predicted values were extracted from a Brazilian population study^22^.

To determine miRNAs expression, we performed miRNA extraction, synthesis of cDNA and samples quality control and PCR array analyses, as follows:

### MiRNA extraction

To determine miRNAs expression, we performed miRNA extraction, synthesis of cDNA, and samples quality control and PCR Array analyses. Total miRNA was extracted from 200 μL plasma using the miRNeasy Serum/Plasma Kit (Qiagen, GmbH, Hilden, Germany) as described by the manufacturer. During extraction, 3.5 μL of miRNeasy Serum/Plasma Spike-In Control (1.6 × 108 copies/μl—*Caenorhabditis elegans* miR-39 miRNA mimic) was added to each sample as the internal control. The miRNA was purified in RNeasy Serum/Plasma mini spin column and eluted in 14 μL of RNase-free water. After the procedure, all samples were stored at -80 °C until cDNA synthesis^23^.

### Synthesis of cDNA and samples quality control

First strand cDNA synthesis was performed using miScript II RT kit (Qiagen, GmbH, Hilden, Germany) according to the manufacturer; 9 µl of miRNA sample was used in a total of 20 µl reactions (4 µl of 5 × miScript HiSpec buffer, 2 µl of 10 × miScript Nucleics mix, 2 µl of transcriptase Mix and 3 µl of RNase-free water). The quality control of cDNA samples was performed by miScript miRNA QC PCR array system (MIHS-989ZC-1) using miScript AYBR Green PCR kit (Qiagen, GmbH, Hilden, Germany). The control conditions are the following: C. elegans miR-39, miR-16, miR-21, miR-191, snoRNA (SNORD 61, 95 and 96), negative and positive PCR control. This system permits to check if there are reverse transcription inhibitors, PCR amplification efficiency, and DNA contamination^24^.

### PCR array analyses

The miScript miRNA PCR array system, which analyzes the expression of 84 miRNAs associated with human inflammatory and auto-immune response (MIHS-105ZR), was performed in RotorGene Real-Time PCR system (Qiagen, Hilden, Germany). The PCR cycling condition was set as follows: 1 cycle at 95 °C for 10 min, 40 cycles at 95 °C for 15 s, and 60 °C for the 30 s. The data containing the Ct (cycle of a threshold) values were analyzed by Qiagen web-based PCR array software using the 2^−∆∆Ct^ formula to compare miRNA expression between groups^25^ Ct value higher than 35 indicates no expression of miRNA in the sample and was excluded from the analysis. The global mean normalization method was used to normalize the miRNA Cts^26^. The miRNAs showing ≥ twofold changes and p-value < 0.05 were considered as differentially expressed.

### Statistical analysis

The data analysis for this paper was generated using SAS software, version 9.3 of the SAS System for Windows. Copyright 2011 SAS Institute Inc., Cary, NC, USA.

For the miRNA statistical analysis, we used the Qiagen GeneGlobe Data Analysis Web Research Center with miScript miRNA PCR Arrays & Assays Data Analysis Software (Qiagen, Hilden, Germany), available in https://geneglobe.qiagen.com/br/analyze/ on November 15th, 2019. We performed descriptive analyses and expressed continuous variables as mean ± SD or median (25–75%), and categorical variables were expressed as absolute numbers or proportions. Demographic and clinical characteristics, exhaled CO, and STT data comparisons between the two groups were performed using the Kruskall–Wallis test or Chi-Square test, when appropriate. Plasma miRNAs expression levels for pre and post periods were analyzed using paired T-test, and between groups were analyzed using unpaired T-test. Correlations between variables were performed using Spearman Coefficient Correlation. Statistical significance was defined as a two-tailed p-value < 0.05. Graphics for Fig. 2 were performed with Graph Pad Prism version 5 Software (California Corporation, California, USA).

## Results

**Figure 1:**
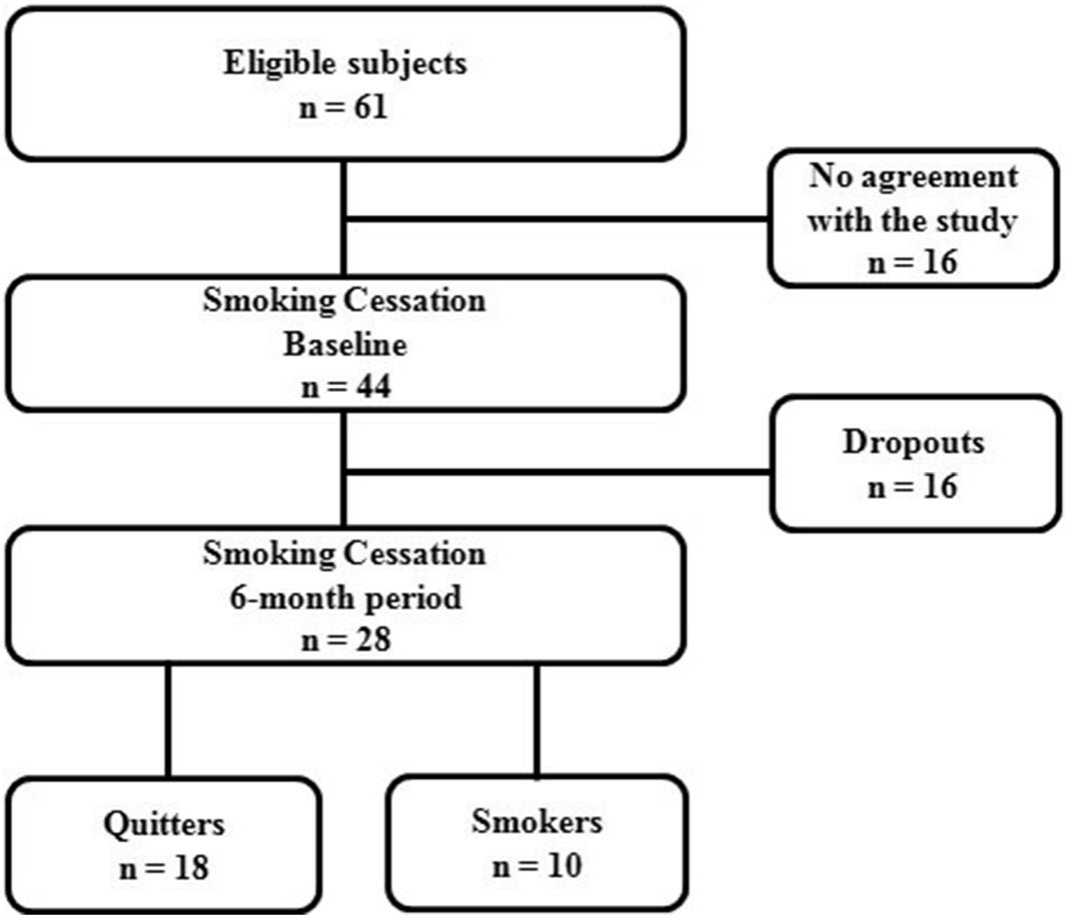
Study flowchart.

Sixty-one individuals from the Smoking Cessation Program of Dante Pazzanese Institute of Cardiology of São Paulo State were invited to participate in this study, between August 2015 and October 2017. Only 44 individuals agreed to participate and were included in this longitudinal study. Sixteen individuals dropped out of the program over the 6-month time period (Fig. 1). Of 28 individuals, 18 (73.7%) succeeded in quitting smoking (100% reduction) and 10 (26.3%) failed to quit, but they reduced smoking load (mean reduction of 58.5 ± 21.2%, varying between 20 and 90%).

At Baseline, analysis of demographic and clinical characteristics, including smoking history (number of cigarettes per day and number of years smoked), showed that Smokers and Quitters were homogeneous groups (Table 1).

**Table 1:** Demographic characteristics and clinical data in quitters and smokers at baseline.

	Quitters n = 18	Smokers n = 10	*p*-value
**Age** (years)	57 ± 7	52 ± 7	0.123
**Male** [n (%)]	11 (61)	3 (30)	0.237
**BMI** (kg/m^2^)	27.0 ± 4.6	30.2 ± 8.2	0.270
**Smoking history**			
Pack years	43.7 ± 18.0	41.2 ± 20.0	0.532
Cigarettes per day	22 ± 7	23 ± 10	0.816
Years of smoking	39 ± 10	36 ± 9	0.428
**Other morbidities [n (%)]**			
Hypertension	16 (89)	9 (90)	0.927
Diabetes	4 (22)	4 (40)	0.318
Dyslipidemia	11 (61)	5 (50)	0.569
COPD	4 (22)	2 (20)	0.890
Emphysema	0 (0)	1 (10)	0.171
Depression	2 (11)	0 (0)	0.274
**Vital signs**			
Systolic blood pressure (mmHg)	138 ± 28	130 ± 28	0.259
Diastolic blood pressure (mmHg)	86 ± 23	78 ± 16	0.179
Heart rate (bpm)	70 ± 12	78 ± 12	0.072
Respiratory rate (rpm)	13 ± 2	14 ± 1	0.347

BMI, body mass index; COPD, chronic obstructive pulmonary disease.

The number of smoked cigarettes per day was significantly different between groups (Table 2). The exhaled CO levels and STT values were similar between groups at Baseline. Quitters had both variables reduced significantly after 6 months. No significant differences were observed in peripheral oxygenation and lung function at Baseline and after 6 months.

**Table 2:** Comparative data (mean ± SD) analysis of number of smoked cigarettes per day, exhaled CO, STT, peripheral oxygenation, and lung function values between the groups at Baseline and after 6 months. a, p < 0.001 versus baseline; b, p < 0.001 versus smokers at the same time; c, p = 0.002 versus baseline; d, p = 0.020 versus baseline; e, p = 0.040 versus smokers at the same time.

	Quitters n = 18		Smokers n = 10	
	Baseline	6-month	Baseline	6-month
Cigarettes per day (n)	22 ± 7	0 ± 0^a,b^	23 ± 10	10 ± 10^c^
Exhaled CO (ppm)	10 ± 9	3 ± 2^a,b^	13 ± 6	8 ± 3^d^
STT (min)	16.0 ± 12.0	8.5 ± 8.0^d,e^	23.0 ± 19.0	16.0 ± 16.1
Peripheral oxygenation (%)	95 ± 2	96 ± 1	94 ± 2	94 ± 2
FVC (L)	3.41 ± 0.87	3.37 ± 0.86	3.32 ± 1.10	3.20 ± 1.00
FVC pred (%)	89.2 ± 12.2	86.4 ± 14.1	90.3 ± 18.3	87.6 ± 19.0
FEV_1_ (L)	2.45 ± 0.67	2.38 ± 0.64	2.38 ± 0.78	2.52 ± 0.90
FEV_1_ pred (%)	81.0 ± 14.2	78.0 ± 15.0	80.0 ± 18.0	85.6 ± 21.0
FEV_1_/FVC ratio	0.72 ± 0.07	0.71 ± 0.07^b^	0.71 ± 0.11	0.78 ± 0.06

STT, saccharine transit time test; FVC, forced vital capacity; pred, predicted; FEV_1_, forced expiratory volume in one second.

We found significant correlations between the number of smoked cigarettes per day and exhaled CO (r = 0.61 and p < 0.001), as between the number of smoked cigarettes per day and STT (r = 0.48 and p < 0.001) and between exhaled CO and STT (r = 0.32 and p = 0.016).

**Figure 2:**
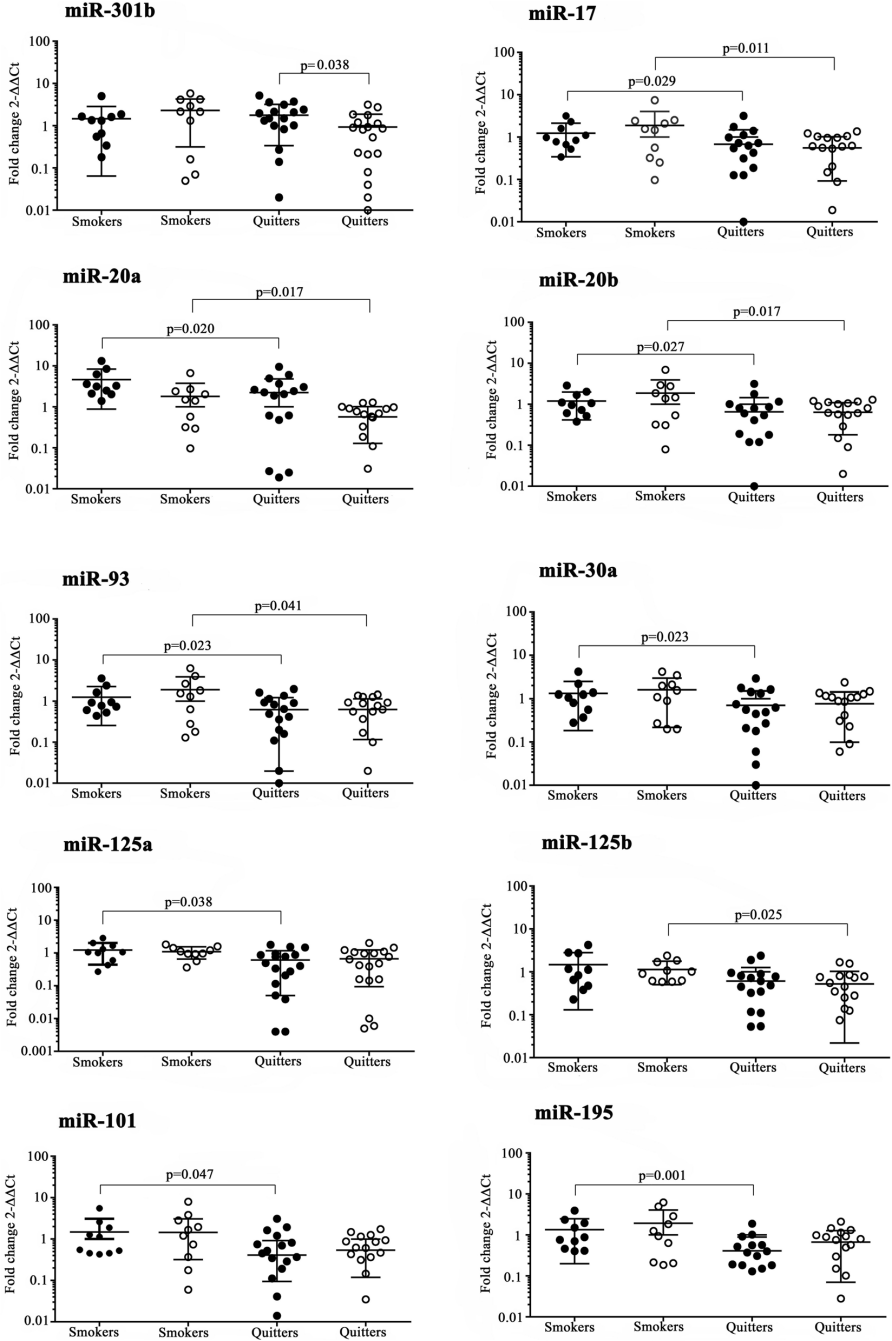
Plasma miRNAs that were differently expressed between smokers and quitters at Baseline (•) and after at 6-month period (ο) as between time-period.

From 84 miRNAs of inflammation and auto-immunity PCR array (Fig. 2), comparisons between groups showed that at baseline, quitters showed significant downregulation of seven miRNAs: miR-17 (− 2.90-fold, p = 0.029), miR-20a (− 3.80-fold, p = 0.021); miR-20b (− 4.71-fold, p = 0.027); miR-30a (− 3.95-fold, p = 0.024); miR-93 (− 3.63-fold, p = 0.022); miR-125a (− 1.70-fold, p = 0.038); and miR-195 (− 5.37-fold, p = 0.002). After 6-month of follow-up, quitters showed significant downregulation in miR-17 (− 5.30-fold, p = 0.012), miR-20a (− 2.04-fold, p = 0.017), miR-20b (− 5.44-fold, p = 0.017), miR-93 (− 4.00-fold, p = 0.041), miR-101 (− 4.82-fold, p = 0.047) and miR-125b (− 3.65-fold, p = 0.025). Among those miRNAs, five were commonly downregulated in quitters: miR-17-5p, miR-20a-5p, miR-20b-5p, miR-93-5p and miR-125. Comparisons between Baseline and 6 months showed that smokers did not change miRNA profile and quitters had one miRNA: miR-301b-3p that was negatively expressed (− 2.29-fold, p < 0.038).

## Discussion

This study provides new information on miRNA profiles of smokers enrolled in smoking cessation programs after 6 months. First, plasma miRNA profiles were heterogeneous among heavy smokers (≥ 15 smoked cigarettes per day) between those who succeed and who failed to quit smoking even with similarities in demographic and clinical characteristics. A mean 50% reduction in the number of smoked cigarettes was insufficient to change the miRNA profile of smokers, and only quitting smoking significantly downregulated miR-301b after 6 months.

Tobacco smoking promotes inhalation of more than 9,000 chemical substances that, in turn, possess cytotoxic, mutagenic, pro-inflammatory, and auto-immune-regulatory properties inducing hypoxic conditions, inflammatory disorders, and other diseases. Among those substances, CO has a pivotal role in tissue hypoxia in smokers by elevating the production of carboxyhemoglobin^27^. In the present study, exhaled CO was used to confirm the smoking load, as used by others^5,9^. At Baseline, all subjects showed high levels of exhaled CO (~ 10 ppm). After 6 months, as expected, quitters decreased exhaled CO levels at a range of non-smokers^9,16^, and smokers group reduced exhaled CO to the level of light smokers (~ 10 cigarettes per day)^28^. Besides exhaled CO, we accompanied smoking cessation with the aid of a functional airway primary defense marker, the STT. Both variables were significantly correlated with the number of smoked cigarettes per day. In the beginning, all subjects showed impaired nasal mucociliary clearance observed by the prolonged STT^5,20^ and significant improvements were observed after smoking cessation, that are following others^5^. Interestingly enough, the present study showed that only quitting smoking reduced STT values to the normal range (≤ 12 min), that may be associated with the respiratory epithelium restoration^29,30^.

Research on circulating miRNAs has been developed, aiming to discover new potential markers to facilitate the early and non-invasive diagnosis of diseases and possible treatment methods using mimetic miRNAs and miRNA inhibitors^31^. The miRNA miR-301b is associated with multiple pathological conditions and can be raised as a non-specific biomarker. However, studies showed its potential role as a biomarker of lung cancer^32,33^. In an interesting study^32^ several experiments with lung cancer cells and animals analyzed the possible mechanism of hypoxic conditions participating in lung cancer. They found that lung cancer cells, human patient tissues, and cells treated under hypoxia environment, both type cells presented increased expression of HIF1-α (hypoxia-inducible factor 1 alpha) protein that in turn increased the expression of miR-301b. The high expression of miR-301b supressed the expression of Bim, a pro-apoptotic protein. In additional experiments in vivo nude mice, the expressions of HIF-1α and miR-301b also increased, and Bim expression decreased, resulting in tumor growth and impairment in programmed cell death. They concluded that high expression of miR-301b could be associated with cell proliferation, decreases in apoptosis, development, and progression of cancer, and increases in resistance to chemotherapy as in metastasis. Therefore, miR-301b can be a potential target for chemotherapy in lung cancer. In line with this work, other studies showed that solid tumors have cell characteristics of hypoxic conditions and autophagy associated with the miRNAs profile’s dysregulation^34,35^. We did not measure tissue hypoxia as hypoxia has a pivotal role in cancer development and progression (prostate, colorectal, pancreas, oral cavity, and lung carcinomas)^36^. However, more importantly, we succeeded in showing that plasma miR-301b expressions were similar between groups at Baseline when subjects are heavy smokers and that miR-301b was significantly downregulated in subjects who quitted smoking after 6 months.

The condition of hypoxia due to exposure to carbon monoxide in smokers has well defined in the literature^27,37^ and smoking is the leading risk factor for the development of lung cancer^31,38^. In clinical practice, late diagnosis of lung cancer has been performed in 60% of the patients leading to a low survival rate after 5 years. The use of feasible methods with more in-depth knowledge of genetic and epigenetic tumorigenesis aspects should be considered^31^. Our study showed that quitting smoking-induced to a clinical endpoint improvement on the mucociliary clearance, an important primary airway defense mechanism, probably because of the restoration of the respiratory epithelium^29,30,39^. The two primary smoking-related diseases, COPD and lung cancer, have common mechanisms for their development, including inflammation, epithelial-mesenchymal transition, oxidative stress, altered DNA repair, angiogenesis and cell proliferation / anti-apoptosis. The conditions are also present related to deregulation of miRNA expression^40,41^. Furthermore, studies show the relationship between the hypoxia condition promoted by tobacco exposure and the expression of HIF-1α and the development of COPD^42,43^. We also showed the miR-301b expression was useful to determine who quit smoking and who continue to smoke. The use of miR-301b as a biomarker of the early onset of COPD and lung cancer still needs further validation in cells/tissue and investigation in larger groups of subjects over longer period of follow-up.

Our study has some limitations. The majority of the miRNA studies use tissue of different organs and/or cell culture. We use plasma as a substrate for miRNAs profile determination as miRNAs are expressed in several loci with a wide and complex circulatory net. Additionally, circulating miRNAs are stable and may resemble tissue miRNA expression. We did not include a healthy group and a validating study with lung tissue. However, we succeeded in showing different plasma miRNA profiles between who quit smoking and who failed after 6 months. Another aspect is that one could consider our sample size as reduced. However, clinical longitudinal studies are limited. A nice study^44^ using a panel of 662 miRNAs showed that 11 smokers had overexpression of 44 plasma miRNA compared with 7 non-smokers. After a 1-month period of smoking cessation, 4 persons showed downregulation of several miRNAs, including some miRNAs that were also downregulated in our study’s quitters after 6 months, particularly miR-17, miR-20 and miR-93. Indeed, our present longitudinal study showed significant differences in plasma miRNA profile between persons who succeed and who failed in both periods of the study: at Baseline and after 6 months. Compared with smokers, quitters showed significant downregulation of important miRNAs (miR-17, miR-20a, miR-20b, miR-93 and miR-125) enrolled in nasopharyngeal carcinogenesis^45^, lung cancer^46–49^, metastasis and cisplatin-resistance^50^, when over-expressed through different signal pathway-mediated mechanisms. These differences in plasma miRNA profiles between the two groups remained after 6 months. None of the subjects was diagnosed with cancer, and only 20% of subjects were diagnosed with COPD in each group. We raised the possibility that dowregulation of miRs 17, 20, 93 and 125 in quitters, despite similarities in demographic, clinical characteristics and smoking history with smokers, maybe related to a possible non-reported smoking load at the beginning of this study and the total abstinence at the end of the study. All miRNAs changes may have possible benefits for the prevention of lung diseases and cancer^14,48,49^.

One could argue that we used only the cut-off values of CO ≤ 6 ppm to identify nonsmoking status. However, we followed the national Guidelines for smoking cessation programs^16^ as in line with others that have used similar cut-off values (≤ 6.5 ppm) with a sensitivity of 90% and a specificity of 83% to identify non-smokers^51^. A nice review^52^ concluded that the cotinine analysis for abstinence detection can be replaced by the association of self-reports of smoking cessation and exhaled CO bellow the cut-off values. Additionally, the use of cotinine analysis to identify non-smokers and smokers can be affected by nicotine replacement as pointed out by others^30^.

In the present study, we did not observe any significant clinical changes over the study period as well as in lung function, possibly because individual demographic and clinical characteristics may affect outcomes after smoking cessation, one person is more resistant to improvement than others, as previously pointed out^53^. Other aspects could have contributed to these results, such as the use of medications, morbidities, and the period of follow-up. The first-line pharmacological treatment for smoking cessation in most countries is varenicline and nicotine replacement combined with cognitive-behavioural therapy, the combination of therapies are more effective than isolated drugs in smokers, with depression and smokers with chronic obstructive pulmonar disease^54–56^. In the present study, we used bupropion and nicotine replacement combined with counseling according to Brazilian guidelines^57^ and Brazilian Ministery of Health financial support. In our study, there were no significant differences in clinical assessments and history or medications between the two groups. Other smoking cessation studies showed no changes in nasal inflammation after a 12-month period^5^ but significant improvements in lung function and inflammation after a 24 months^58^.

In conclusion, reductions in the number of smoked cigarettes were insufficient to change plasma profile of miRNA in 6 months. Only quitting smoking (100% reduction) significantly downregulated miR-301b related to hypoxic conditions, promotion of cell proliferation, decreases in apoptosis, cancer development, and progression as increases in radiotherapy and chemotherapy resistance.
